# Supplementary material for: The Systems Biology Research Tool: evolvable open-source software
Source: BMC Syst Biol. 2008 Jun 29;2:55. doi: 10.1186/1752-0509-2-55 (PMC2446383; doi:10.1186/1752-0509-2-55)
Supplement: Additional file 1 — SBRT Archive. An archive of the current version of the Systems Biology Research Tool. [file 1752-0509-2-55-S1.zip › sbrt-1.4.0/doc/developers_guide/api/sbrt/shell/text/chem/package-summary.html]

sbrt.shell.text.chem


|  |  |  |
| --- | --- | --- |
| **Overview** \| **Package** \| Class \| **Use** \| **Tree** \| **Deprecated** \| **Index** \| **Help** | |  |
| **PREV PACKAGE**   **NEXT PACKAGE** | **FRAMES**    **NO FRAMES**     **All Classes** |  |


---

## Package sbrt.shell.text.chem

Provides classes and interfaces for defining formats for chemistry related
objects.

**See:**
  
          **Description**

| **Interface Summary** | |
| --- | --- |
| **CatalystFormat** | This interface is used to represent the formats of catalysts in chemical reactions. |
| **ChemSpeciesFormat** | This interface is used to represent the formats of chemical species. |
| **RxnNameFormat<T>** | This interface is used to represent formats for the names of chemical reactions. |

| **Class Summary** | |
| --- | --- |
| **CatalystFormatV1** | This class is a concrete implementation of `CatalystFormat`. |
| **CatalystFormatV2** | This class is a concrete implementation of `CatalystFormat`. |
| **ChemSpeciesFormatV1** | This class is a concrete implemenation of `ChemSpeciesFormat`. |
| **IrrevRxnFormatV1** | This class is used to format irreversible chemical reactions. |
| **IrrevRxnFormatV2** | This class is used to format irreversible chemical reactions. |
| **RevRxnFormatV1** | This class is used to format reversible chemical reactions. |
| **RevRxnFormatV2** | This class is used to format reversible chemical reactions. |
| **RxnNameFormatV1** | This class is a concrete implementation of `RxnNameFormat`. |

## Package sbrt.shell.text.chem Description

Provides classes and interfaces for defining formats for chemistry related
objects.

---


|  |  |  |
| --- | --- | --- |
| **Overview** \| **Package** \| Class \| **Use** \| **Tree** \| **Deprecated** \| **Index** \| **Help** | |  |
| **PREV PACKAGE**   **NEXT PACKAGE** | **FRAMES**    **NO FRAMES**     **All Classes** |  |


---
